# The Future of Antiarrhythmic Drug Therapy: Will Drugs Be Entirely Replaced by Procedures?

**DOI:** 10.14797/mdcvj.1185

**Published:** 2022-12-06

**Authors:** Miguel Valderrábano

**Affiliations:** 1 Houston Methodist DeBakey Heart & Vascular Center, Houston Methodist Hospital, Houston, Texas, US

**Keywords:** ethanol, ablation, ventricular vein, ventricular arrhythmias

## Abstract

Antiarrhythmic drug therapy has traditionally been centered in modulating the generation or propagation of the cardiac action potential by drugs acting on membrane ion channels. The history of this approach has been disappointing, marked by catastrophic failures such as those of sodium channel blockers or sotalol to treat ventricular arrhythmias in the setting of structural cardiomyopathies, which led to increased mortality, and by modest clinical efficacy in paroxysmal atrial fibrillation. As catheter ablation has become an established effective therapy for most tachyarrhythmias, membrane-acting drugs have been relegated to symptomatic control of benign arrhythmias in normal hearts or to adjunctive treatments of ventricular tachycardia (combined with catheter ablation and cardiac defibrillators) in the setting of cardiomyopathies. Novel targets of biological modulation of arrhythmia substrates beyond the membrane potential appear promising and could represent future opportunities for arrhythmia pharmacotherapy.

## Introduction

**Figure 1:**
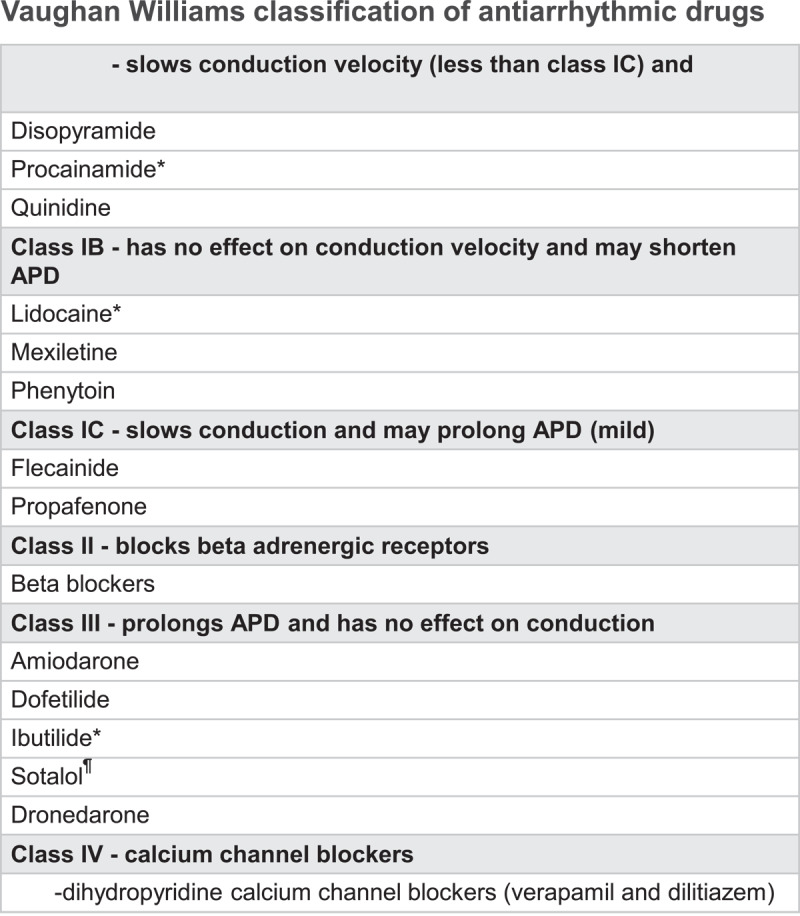
The Vaughan Williams classification provided a framework for understanding antiarrhythmic drugs, which targeted the different molecular components of the membrane action potential. APD: action potential duration

Targeting the membrane potential seemed like a logical foundation to antiarrhythmic therapy. The Vaughan Williams (VW) classification^1,2,3^ provided a framework of understanding of a group of drugs that targeted the different molecular components of the action potential (Figure 1). Beta-blockers (Class II) were an exception, but their inclusion as antiarrhythmics had a wealth of support as suppressors of adrenergic-dependent arrhythmia. The classification was confusing, incomplete, and inconsistent. It grouped drugs by phenomena (conduction velocity, refractoriness) rather than molecular targets, so it was neither clinically relevant nor mechanistically precise. The Sicilian Gambit,^4^ devised 20 years later, attempted to add molecular precision to the classification, only to become clinically unmanageable and irrelevant.

## Historic Failures of Antiarrhythmic Drugs

Clinicians needed clarity as to (1) which drug to use, (2) for which arrhythmia, and (3) in which patient. Clarity came first from negative answers. Proarrhythmia and increased mortality were noted with Class I drugs (eg, sodium channel blockers encainide, flecainide, and moricizine) in patients with post-infarction ventricular extrasystoles as shown in the CAST-1 and CAST-2 trials.^5,6^ D-sotalol, a Class III drug potassium channel blocker, increased mortality in the SWORD trial,^7^ which studied post-infarction patients with ejection fraction < 40%. In the CASH trial, survivors of cardiac arrest treated with propafenone had increased mortality compared with those treated with an implantable cardioverter defibrillator.^8^ Most recently, dronedarone in patients with permanent atrial fibrillation (AF) also increased mortality.^9^

## Remaining Clinical Use of Antiarrhythmic Drugs

**Figure 2:**
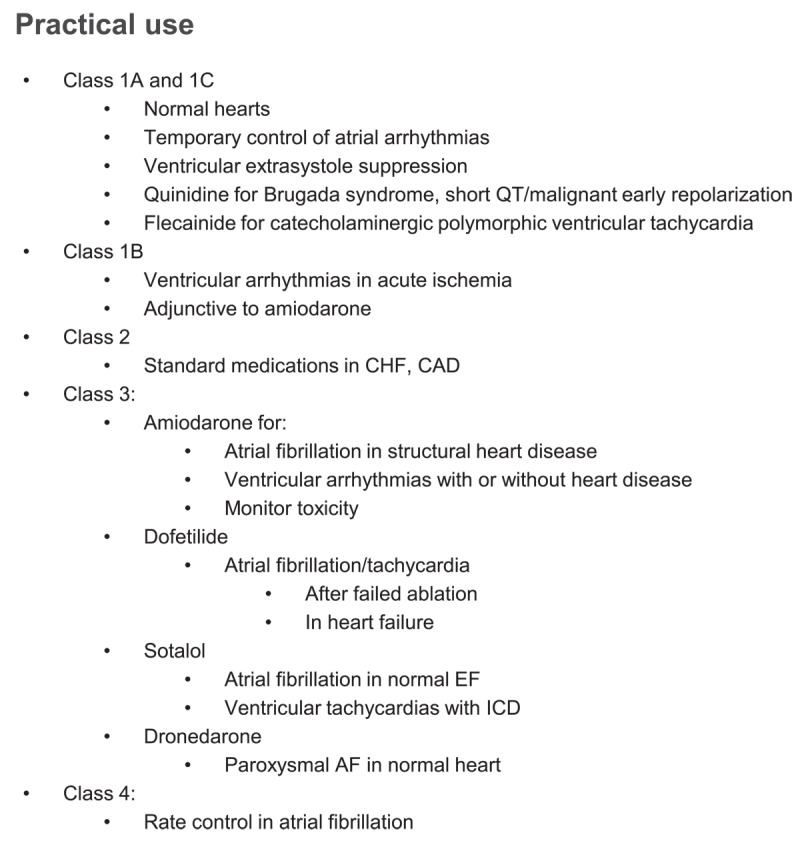
Practical use for antiarrhythmic drugs. CHF: congestive heart failure; CAD: coronary artery disease; EF: ejection fraction; ICD: implantable cardioverter defibrillator; AF: atrial fibrillation

A practical use of antiarrhythmic drugs is outlined in Figure 2. Class IA and IC drugs are safe and well tolerated in normal hearts, free from prior myocardial infarction or from left ventricular dysfunction. They are useful in AF in such patients and are occasionally used for failed ablations of supraventricular tachycardias. Furthermore, they are effective extrasystole suppressors and are used in patients who failed or refused ablation for ventricular extrasystoles. Concerns remain when prescribed for AF without proper rate control since conduction velocity slowing may lead to slow atrial flutter with paradoxical increase in ventricular response. Dronedarone and sotalol remain valid options for nonpermanent AF in the absence of structural heart disease, and coronary artery disease is an acceptable comorbidity for patients treated with sotalol. Dofetilide is an acceptable choice for AF even in heart failure.^10^ Although most clinicians may use drugs as their first choice for rhythm control in AF compared with ablation, emerging data support ablation as first line of therapy, and ablation is undisputedly superior in previous drug failure.^11,12,13^

Special situations of interest include the use of quinidine for suppression of ventricular arrhythmias in Brugada syndrome.^14^

Amiodarone is the most potent antiarrhythmic drug.^15,16^ However, lung, liver, thyroid, eye, and skin toxicities offset its clinical benefits and lead to the need for patient monitoring, requiring periodic toxicity monitoring.^17^ Thus, management guidelines recommend the use of amiodarone “only after consideration of risks, and when other agents have failed or are contraindicated.”^18^ Currently it is used for AF and for ventricular tachycardia in the context of structural heart disease.

A particularly complex situation arises when patients with significant cardiomyopathy develop ventricular arrhythmias requiring defibrillator shocks. In this scenario, amiodarone is a commonly used drug. Sotalol can decrease defibrillator shocks. Drug combinations including mexiletine can be effective. Catheter ablation can lead to improved outcomes rather than escalating antiarrhythmic drugs.^19^

## Unclassified Drugs: Ivabradine, Nasal Etripamil

Developed after the VW classification, ivabradine acts on the *I*_f_ current, present in pacemaking cells of the sinus and atrioventricular nodes, with the chief effect of slowing the heart rate. Although ivabradine is approved by the US Food and Drug Administration (FDA) for the treatment of heart failure,^20^ its main clinical use is for the treatment of symptomatic inappropriate sinus tachycardia.^21^

Although not FDA approved, etripamil is a calcium channel blocker (thus VW Class IV) that is delivered via nasal spray for the acute termination of supraventricular tachycardia.^22^

## Antiarrhythmic Benefits of Treating Underlying Left Ventricular Dysfunction: “Upstream” Therapies

Most life-threatening arrhythmias arise in the context of some form of heart disease, which determines both the prognostic implication as well as the specific drug treatment. Thus, it is not surprising that treatments targeting underlying left ventricular dysfunction may reduce the incidence of arrhythmias. For example, angiotensin-converting enzyme inhibitors,^23^ beta-adrenergic blockers,^24^ mineralocorticoid receptor antagonists,^25^ sacubitril/valsartan,^26^ and most recently SGLT2 inhibitors^27^ have been shown to reduce arrhythmogenic sudden cardiac death—and, in the case of SGLT2 inhibitors, the incidence of AF,^28^ which was not the case for the other upstream therapies. *Optimized treatment of the underlying heart disease is an integral part of arrhythmia management, more so than any membrane-acting antiarrhythmic drug*.

## The Rise of Catheter Ablation and Device Therapies and Decline of Antiarrhythmics

With improvements in the understanding of cardiac arrhythmia mechanisms, catheter ablation has become the first line of treatment of most supraventricular arrhythmias. Most recently, catheter ablation as a first-line treatment for paroxysmal AF has been shown to have not only improved rhythm control^11,29^ but also reduced progression to persistent AF on long-term follow-up.^13^

Similarly, catheter ablation is a guideline-recommended first-line therapy for ventricular tachycardia (VT) in the setting of ischemic heart disease or nonischemic cardiomyopathy.^30^ Although recent data support ablation early in the course of VT management,^31,32,33^ most centers resort to ablation after failed antiarrhythmic therapy given the aggressive nature of the procedure, which is considered high risk.

## Future Directions

In summary, ablation procedures are at the center of arrhythmia management as potentially curative, mechanistically-driven approaches. Drugs targeting underlying cardiomyopathic processes are mandatory. Antiarrhythmic drugs are relegated to adjuvant or palliative roles.

In terms of future directions, the role of neuromodulatory therapies targeting the cardiac autonomic system is rapidly emerging. This is an opportunity for novel drug targets as much as it is for novel procedural approaches. Gene therapy targeting potential mediators of electrical and neural mediators of AF has shown promising results in preclinical models.^34^

## Key Points

- Novel targets of biological modulation of arrhythmia substrates beyond the membrane potential appear promising and could represent future opportunities for arrhythmia pharmacotherapy.
- Because most life-threatening arrhythmias arise in the context of some form of heart disease, *optimized treatment of the underlying heart disease is an integral part of arrhythmia management, more so than any membrane-acting antiarrhythmic drug*.
- Catheter ablation has become the first line of treatment of most supraventricular arrhythmias. As a first-line treatment for paroxysmal atrial fibrillation (AF), it has been shown to have improved rhythm control and reduced progression to persistent AF on long-term follow-up. In addition, it is a guideline-recommended first-line therapy for ventricular tachycardia in the setting of ischemic heart disease or nonischemic cardiomyopathy.
- Ablation procedures are at the center of arrhythmia management as potentially curative, mechanistically-driven approaches. Drugs targeting underlying cardiomyopathic processes are mandatory. Antiarrhythmic drugs are relegated to adjuvant or palliative roles.
